# Regulation of Fatty Acid Metabolism and Inhibition of Colorectal Cancer Progression by Erchen Decoction

**DOI:** 10.1155/2023/9557720

**Published:** 2023-04-10

**Authors:** Linghong Liao, Fei Zhang, Zewei Zhuo, Chengbao Huang, Xiaofang Zhang, Ruifang Liu, Bizhen Gao, Shanshan Ding

**Affiliations:** ^1^Fujian University of Traditional Chinese Medicine, Fuzhou, Fujian 350122, China; ^2^Fujian Key Laboratory of TCM Health State, Fujian University of Traditional Chinese Medicine, Fuzhou, Fujian 350122, China

## Abstract

Erchen decoction (ECD) is a traditional Chinese prescription widely used in the treatment of various diseases such as obesity, fatty liver, diabetes, and hypertension. In this study, we investigated the effect of ECD on fatty acid metabolism in a colorectal cancer (CRC) mouse model fed a high-fat (HF) diet. The HF-CRC mouse model was established by azoxymethane (AOM)/dextran sulphate sodium (DSS) combined with a high-fat diet. Mice were then gavaged with ECD. Change in the body weight was recorded every two weeks for 26 weeks. Changes in blood glucose (GLU), total cholesterol (TC), total triglycerides (TG), and C-reactive protein (CRP) were measured. Colorectal tissues were collected to observe changes in colorectal length and tumorigenesis. Hematoxylin-eosin (HE) staining and immunohistochemical staining were performed to observe changes in intestinal structure and inflammatory markers. Fatty acids and the expression of related genes in colorectal tissues were also studied. ECD gavage inhibited HF-induced weight gain. CRC induction and HF diet intake resulted in increased GLU, TC, TG, and CRP, where ECD gavage reduced these elevated indicators. ECD gavage also increased colorectal length and inhibited tumorigenesis. HE staining revealed that ECD gavage suppressed inflammatory infiltration of colorectal tissues. ECD gavage suppressed the fatty acid metabolism abnormalities caused by HF-CRC in colorectal tissues. Consistently, ECD gavage lowered ACSL4, ACSL1, CPT1A, and FASN levels in colorectal tissues. *Conclusions*. ECD inhibited HF-CRC progression through the regulation of fatty acid metabolism.

## 1. Introduction

Colorectal cancer is one of the main causes of cancer-related deaths worldwide and is the third most common cancer in both males and females. The International Agency for Research on Cancer estimates that CRC accounts for approximately 10% of all new cancer cases and 9% of all cancer-related deaths [1]. Accordingly, CRC is the third most common cancer worldwide for both male and female, and has high mortality of 45%, 35%, and 47.8% in Europe, the USA, and worldwide, respectively, [2]. The occurrence of colorectal cancer is attributed to a variety of risk factors, of which more than half of diagnoses and deaths are attributed to factors such as an unhealthy diet, lack of exercise, being overweight, smoking, and alcohol. All of these are potentially preventable [3]. Current data further support the tumor-promoting effects of a high-fat diet, which is mainly dependent on the complex interaction between the gut microbiota and bile acid metabolism [4].

In addition, the tumor microenvironment (TME) in colorectal cancer is an important factor in the efficacy of targeted cancer therapies [5]. The tumor microenvironment is characterized by an acidic, anoxic, nutrient-deficient, and highly oxidative environment [6]. Due to rapid cell proliferation and insufficient angiogenesis, cancer cells exhibit unique metabolic characteristics through metabolic reprogramming processes [7]. In all metabolic characteristic studies, abnormalities in cancer cell fatty acid metabolism have been increasingly recognized [8]. Fatty acids are necessary for cell energy storage, membrane proliferation, and signaling molecule generation. Understanding the metabolic mechanisms of fatty acids may promote understanding of the characteristics of TME infiltration, thus guiding the effective treatment of CRC. As previously mentioned, fatty acids affect cancer cell survival, proliferation, and invasiveness in the hypoxic and acidic intestinal microenvironment. Therefore, fatty acids may play a key role in high-fatdiet-induced cancer progression [9].

Traditional Chinese medicine (TCM) has attracted increasing attention due to its potential to enhance the immune system, improve the quality of life, and reduce the side effects of chemotherapy [10]. Erchen decoction (ECD), mainly derived from classic clinical Traditional Chinese Medicine books, is a basic Chinese medicine prescription for the treatment of dryness, dampness, and phlegm. It is widely used to treat various diseases including metabolic disorders induced by ingestion of a HF diet [11]. The main ingredients in ECD include *Pinellia ternata*, *Poria cocos*, *Citrus reticulata*, and *Glycyrrhiza uralensis*. Specifically, *Poria cocos* increased the diversity of the intestinal microbiota and significantly changed the structure and composition of the intestinal microbiota in mice [12]. More importantly, *Poria cocos* has been proven to have antitumor effects in lung cancer and breast cancer [13, 14]. *Glycyrrhiza uralensis* flavonoids are a group of active molecules that have multiple effects on cell growth, survival, and cell signaling [15]. However, the mechanism of the ECD in regulating fatty acid metabolism in colorectal cancer has not been studied. Therefore, this study investigated the effects of ECD on fatty acid metabolism in a colorectal cancer mouse model fed a high-fat diet.

## 2. Materials and Methods

### 2.1. Drug Preparation

All medicines were purchased from the Third People's Hospital Affiliated to Fujian University of Traditional Chinese Medicine (China, Fujian, Fuzhou) (Prescription No.: 1295001). Erchen Decoction was composed of *Pinellia ternata* (15 g), *Citrus reticulata* (15 g), *Poria cocos* (9 g), and liquorice (4.5 g). The medicinal liquid was prepared by filling a medicine pot with the proper amount of cold water and adding the all herbs. The mixture was boiled for 30 min, decocted twice, filtered with six layers of folded sterile gauze, and then concentrated to 0.435 g of crude drug per milliliter with a rotary evaporator (China, Henan, Zhengzhou) and 0.08 Mpa for 30 min. The resulting solution was packed in sterile glass bottles and stored for one week at 4°C and then at −20°C until use.

### 2.2. Experimental Animals

Male ICR mice (five weeks old, weighing 20 ± 2 g) were purchased from Shanghai Leske Experimental Animal Co., Ltd. (SCXK (Shanghai) 2017-0005) (Shanghai, China). The mice were raised in an SPF Animal Room at the Experimental Animal Center of Fujian University of Traditional Chinese Medicine. The laboratory unit license (SYXK (Min) 2014-0001) (Fuzhou, China) was issued. There were four animals per cage, the room temperature was 23°C ± 3°C, the relative humidity was controlled at 50–60%, and the light/dark period was artificially established to provide 12 h of daylight and 12 h of dark. All procedures involving mice in this study were performed in accordance with institutional guidelines, and ethical reviews were provided by the Review Committee of Fujian University of Traditional Chinese Medicine (number 2018-036). The experiment began after a one-week acclimation period.

The ICR mice were divided into four groups: normal group, CRC group, HF-CRC group, and HF-CRC + ECD group (*n* = 8). Beginning in the second week, the mice in the normal and CRC groups were fed ordinary feed fat accounted for 4% of the total calories (1022, Beijing HFK Bioscience Co., Ltd. Beijing, China). Mice in the HF-CRC and HF-CRC + ECD groups were fed the fat accounted for 45% of total calories (D12451, FBH Biotechnology Co., Ltd., Shanghai, China) until week 26. The AOM/DSS four-step method was utilized to induce CRC. The mice were injected intraperitoneally with AOM (diluted to 1 mg/mL with normal saline) at a dose of 0.15 mL/g at week 8. One week after recovery, the mice began drinking 2% DSS for seven days during the first cycle of DSS induction, followed by a four-week recovery period (normal drinking water). Therefore, 35 days were set as one cycle for DSS induction [16]. Starting on the fifth week, ECD gavage (20 mL/kg/d) was carried out once a day until the end of 26 weeks. The mice in other groups were given an equal volume of normal saline. Weight was measured once a week until the study endpoint. Blood samples were drawn after 26 weeks, and the mice were euthanized by intraperitoneal injection of excess pentobarbital sodium (150 mg/kg). Colorectal tissues were collected, the length was measured, and tumors were counted.

### 2.3. Biochemical Analyses

Blood samples were collected and serum was isolated by centrifugation (4°C, 3,000 g, 15 min). Triglycerides (TG), total cholesterol (TC), and C-reactive protein (CRP) levels were detected using ELISA kits according to the manufacturer's instructions.

### 2.4. Hematoxylin-Eosin (HE) Staining

Pathological sections of rectal tissue near the anus were prepared, fixed with 4% paraformaldehyde solution, embedded in paraffin, and cut into a series of 5-*μ*m-thick sections. Paraffin-embedded sections were dewaxed and stained with hematoxylin and eosin (Solarbio, Beijing, China). An optical microscope (Axio ImagerA2, Germany) was used to observe inflammatory infiltration of the tissue.

### 2.5. Fatty Acid Detection

Colorectal tissues were mixed with 100 mg pyrogallic acid, several zeolites, and 2 mL 95% ethanol. After the addition of 10 mL hydrochloric acid solution, the flask was placed in a water bath at 70–80°C for 40 min. The flask was oscillated every 10 min so particles attached to the flask wall were mixed into the solution. After hydrolysis, the flask was cooled to room temperature.

The hydrolyzed samples were mixed well with 10 mL of 95% ethanol. The hydrolysate from the flask was transferred to a split funnel, and the flask and plug were rinsed with 50 mL ether petroleum ether mixture. The flushing fluid was incorporated into the split funnel, and the funnel was capped. The split funnel was oscillated for 5 min and allowed to stand for 10 min. The ether layer extract was collected into a 250 mL flask. In accordance with the abovementioned steps, the hydrolysate was extracted three times, and the funnel was washed with an ether petroleum ether mixture and collected into the flask. The flask was steamed in a water bath and dried in a 5°C oven for 2 h.

The fat extract was placed in a water bath with 2 mL of 2% sodium hydroxide methanol solution for 30 min and then with 3 mL of 14% boron trifluoride methanol solution for 30 min (both at 85°C). Then, the temperature was dropped to room temperature, and 1 mL n-hexane was added to the centrifuge tube, followed by a 2 min oscillation and 1 h of standing for stratification. The supernatant (100 *μ*L) was added with n-hexane to a final volume of 1 mL. After filtering with a 0.45 *μ*m filter membrane, the sample was loaded onto a gas chromatograph mass spectrometer (Trace1310ISQ, Thermo Fisher Scientific Inc., Waltham, MA, USA). The formula used was as follows: *W*=*C∗V∗N*/*m∗k*, where *W* is the content of each fatty acid in the sample in milligrams per kilogram (mg/kg), *C* is the concentration of fatty acid methyl ester in the sample in mg/L, *V* is the volume in mL, *k* represents the conversion coefficient of each fatty acid methyl ester to fatty acid (Table 1), *N* is the dilution multiple, and *m* denotes sample weight in grams (*g*).

### 2.6. Reverse Transcription Quantitative Polymerase Chain Reaction (RT-qPCR) Analysis

Total RNA was extracted from the mouse colorectal tissues using TRIzol reagent and transcribed into cDNA using the Premix Ex Taq™ II kit (Takara Biotechnology Ltd., Dalian, Liaoning, China). mRNA expression was quantified using a Premix Ex Taq™ II kit (Takara Biotechnology Ltd., Dalian, Liaoning, China) on a QuantStudio™ 6 Flex Real-Time PCR System (ABI Company, Oyster Bay, NY, USA). Glyceraldehyde-3-phosphate dehydrogenase (GAPDH) was used as the internal reference gene to obtain the Ct value of the target gene. We used the 2^−ΔΔCt^ method to determine relative mRNA expression. The sequence is presented in Table 2.

### 2.7. Western Blotting

The colorectal tissues were incubated in cold radio immunoprecipitation assay buffer (KaiJi, Jiangsu, China) for 0.5 h at 4°C, centrifuged at 12,000 rpm for 0.5 h, and extracted with a 5 × loading buffer (KaiJi, Jiangsu, China) solution. The lysate was boiled at 100°C for 10 min. Samples were separated by 10% SDS-polyacrylamide gel electrophoresis and transferred to NC membranes (Life Technologies, Carlsbad, CA, USA). After sealing in 5% skim milk for 2 h, the membrane was incubated with a rabbit anti-ACSL4 primary antibody (1:1000, ImmunoWay Biotechnology, Plano, TX, USA) and then with the secondary antibody at room temperature for 1.5 h. The primary antibodies including anti-ACSL4 primary antibody (1 : 1000, ImmunoWay Biotechnology, Plano, TX, USA), rabbit antii-ACSL1 (1 : 1000, ImmunoWay Biotechnology, Plano, TX, USA), rabbit anti-CPT1A (1 : 1000, ImmunoWay Biotechnology, Plano, TX, USA), rabbit anti-Fas (1 : 1000, ImmunoWay Biotechnology, Plano, TX, USA), and rabbit anti-GAPDH (1 : 5000, ImmunoWay Biotechnology, Plano, TX, USA) were similar. Bands were then detected using a chemiluminescence detection kit (Bio-Rad Laboratories, Hercules, CA, USA). Subsequently, Quantity One software (Bio-Rad) was used to analyze the relative expression of bands with GAPDH as the internal control.

### 2.8. Statistical Analysis

The analyses and graphs of all experimental data were carried out with GraphPad Prism 8.0 software (GraphPad, San Diego, CA, USA). The measurement data are expressed as the mean ± standard deviation (SD) of at least three independent experiments. Differences between groups were assessed by one-way or two-way analysis of variance (ANOVA), and Tukey's post hoc test was used for multiple comparisons. *p* < 0.05 was considered indicative of a statistically significant difference.

## 3. Results

### 3.1. ECD-Inhibited Tumor Growth in HF-CRC Mice

**Figure 1:**
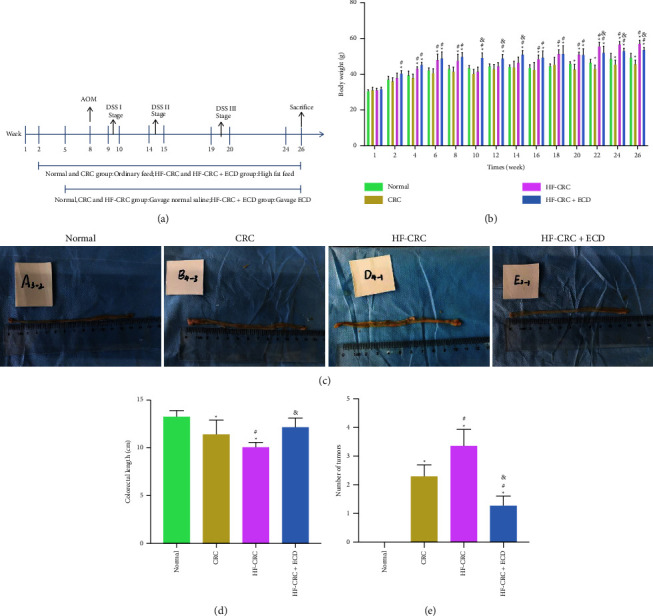
ECD-inhibited CRC tumor growth. (a) Modeling process in mice; (b) weight changes were measured every two weeks; (c) mouse colorectal tissues harvested after 26 weeks; (d) colorectal length in each group; (e) tumor formation in colorectal tissues in each group. Values represent the mean ± SD (*n* = 8). Differences between groups were assessed by one-way or two-way ANOVA, and Tukey's post hoc test was used for multiple comparisons. ^*∗*^*p* < 0.05 vs. normal mice; ^#^*p* < 0.05 vs. CRC mice; ^&^*p* < 0.05 vs. HF-CRC mice.

We established a HF-CRC mouse model using AOM/DSS combined with application of an HF diet (Figure 1(a)). Body weight changes were detected in mice at an interval of two weeks (Figure 1(b)). We observed a gradual increase in body weight in the HF-CRC and HF-CRC + ECD groups beginning in the second week. By the 8th week of AOM intraperitoneal injection, the weight of these two groups of mice was significantly higher than the mice fed with a normal diet. The use of DSS in the first cycle resulted in varying degrees of weight loss in mice (week 10), while ECD gavage significantly inhibited the sharp decrease in body weight. The HF diet gradually led to abnormal weight gain in mice during the subsequent DSS induction cycle, while ECD inhibited abnormal weight gain to some extent.

After 26 weeks, mouse colorectal tissues were harvested and photographed (Figure 1(c)). The tissue length was measured and tumor number was counted (Figures 1(d) and 1(e)). CRC resulted in a shorter colorectal length, and the HF diet exacerbated this colorectal shortening, whereas ECD gavage significantly enhanced colon length in HF-CRC mice. No obvious pathological changes were observed in the tissues of the normal mice. However, CRC induction led to tumor production in the colorectal tissue, which was accompanied by ulcers, and ECD treatment significantly inhibited tumor growth.

### 3.2. Effects of ECD on HF-CRC Mice

**Figure 2:**
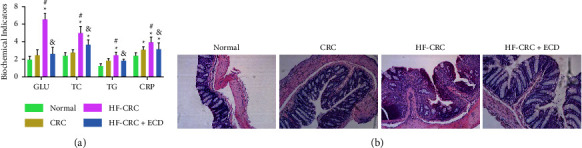
Effects of ECD therapy on biochemical markers and inflammatory infiltration in mice. (a) Biochemical markers in the blood of mice in each group; (b) HE staining for inflammatory infiltration of colorectal tissues. Values represent the mean ± SD (*n* = 8). Differences between groups were assessed by two-way ANOVA, and Tukey's post hoc test was used for multiple comparisons. ^*∗*^*p* < 0.05 vs. normal mice; ^#^*p* < 0.05 vs. CRC mice; ^&^*p* < 0.05 vs. HF-CRC mice.

Finally, we analyzed the biochemical indices of the mice (Figure 2(a)). CRC modeling resulted in an increase in GLU, TC, TG, and CRP levels in mice, while the HF diet further promoted this increase. ECD gavage significantly inhibited the increase of GLU and TC in HF-CRC mice and reduced TG levels to some extent, indicating that ECD affects fatty acid metabolism. In addition, ECD gavage significantly inhibited serum CRP levels in HF-CRC mice, indicating a possible correlation between ECD gavage and inflammation. Next, we examined whether ECD treatment affected inflammatory infiltration in colorectal tissues via HE staining (Figure 2(b)). Results showed that in the normal group, the intestinal mucosal cells and recesses were normal, the intestinal glands were completely arranged, the villus structure was intact and arranged normally, and no tumors were observed. In the CRC group, the connective tissue of the large intestine was loose, the intestinal glands were arranged loosely, and there were chronic inflammatory changes. Some tumor cells had formed glandular tubes of varying sizes, and the tumor cells were arranged in a columnar shape. There were more inflammatory cells infiltrating the interstitial connective tissue of the glandular tubes. In the HF-CRC group, the intestinal mucosal crypt structure was disordered and contained abnormal cells, large and deep stained nuclei, pathological mitosis, high-grade intraepithelial neoplasia, local mucosal carcinoma formation, tumor cells with significant differences in cell size and morphology, and a large number of inflammatory cells infiltrating the stroma. The colon tumors in the HF-CRC + ECD group were mainly low-grade intraepithelial neoplasia, and the infiltration of inflammatory cells was mild.

### 3.3. Effects of ECD on Fatty Acid Metabolism

**Figure 3:**
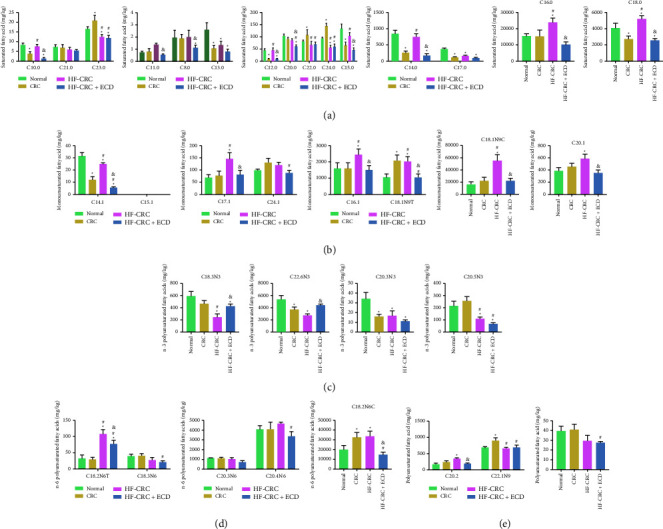
Effects of ECD on fatty acid metabolism in HF-CRC mice. (a) Changes in saturated fatty acid metabolism; (b) metabolic changes in monounsaturated fatty acid metabolism; (c) changes in *n* − 3 polyunsaturated fatty acid metabolism; (d) metabolic changes in *n* − 6 polyunsaturated fatty acid metabolism; (e) changes in polyunsaturated fatty acid metabolism. Data are presented as the mean ± SD of three independent experiments. Differences between groups were assessed by one-way or two-way ANOVA, and Tukey's post hoc test was used for multiple comparisons. ^*∗*^*p* < 0.05 vs. normal mice; ^#^*p* < 0.05 vs. CRC mice; ^&^*p* < 0.05 vs. HF-CRC mice.

To investigate the specific effects of ECD on fatty acid metabolism, we measured fatty acids in the colorectal tissues of mice in each group. Fatty acids at a level of <0.05 mg/kg were defined as undetected. We classified the results as saturated fatty acids (Figure 3(a)), monounsaturated fatty acids (Figure 3(b)), *n* − 3 polyunsaturated fatty acids (Figure 3(c)), *n* − 6 polyunsaturated fatty acids (Figure 3(d)), and polyunsaturated fatty acids (Figure 3(e)). We observed that fatty acid metabolism was abnormal due to CRC or HF-CRC induction, and ECD significantly improved fatty acid metabolism. ECD promoted the synthesis of *n* − 3 polyunsaturated fatty acids such as C22:6n3 (Cis-4,7,10,13,16,19-docosahexaenoic acid) and C18:3n3 (*α*-linoleic acid) and repressed the synthesis of *n* − 6 polyunsaturated fatty acids such as C18:2n6c (linoleic acid).

### 3.4. Effects of ECD on the Expression of Genes Related to Fatty Acid Metabolism

**Figure 4:**
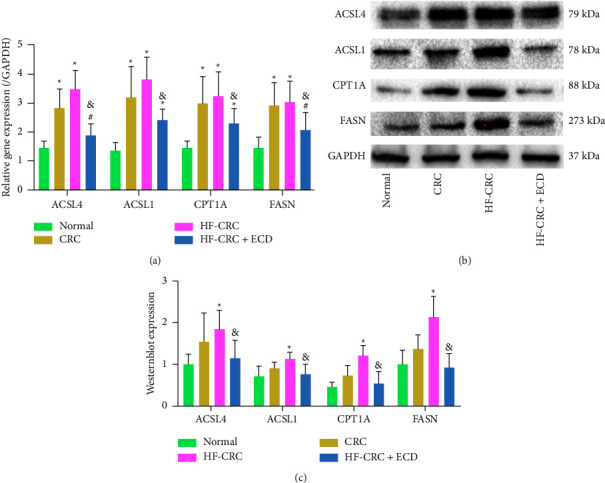
Effects of ECD therapy on fatty acid metabolism-related gene expression. (a) The mRNA expression of fatty acid metabolism-related genes in HF-CRC mice as examined by RT-qPCR; (b, c) the protein expression of fatty acid metabolism-related genes in HF-CRC mice as examined by Western blotting. Data are presented as the mean ± SD of three independent experiments. Differences between groups were assessed by two-way ANOVA, and Tukey's post hoc test was used for multiple comparisons. ^*∗*^*p* < 0.05 vs. normal mice; ^#^*p* < 0.05 vs. CRC mice; ^&^*p* < 0.05 vs. HF-CRC mice.

Furthermore, we detected changes in fatty acid metabolism in mice at the gene level, including the expression of ACSL4, ACSL1, CPT1A, and FASN. Through RT-qPCR, we observed that CRC induction led to an increase in the expression of the four genes, and the HF diet also promoted their expression to some extent. However, ECD gavage significantly downregulated all four genes compared with the HF-CRC group, but not ACSL1 and CPT1A when compared with the CRC group (Figure 4(a)). Western blotting showed consistent results (Figures 4(b) and 4(c)).

## 4. Discussion

CRC is a condition caused by complex interactions between gene susceptibility, clinical conditions, and environmental/lifestyle-associated risk factors such as smoking, alcohol consumption, a HF diet, and overweight. The hypothesis that an HF diet and related metabolic biomarkers play an important role in cancer etiology has been supported [17]. The main components of each medicine in ECD contain bioactive components including flavonoids (e.g., liquiritin, liquiritigenin, and hesperidin), triterpenoids (e.g., glycyrrhizin and pachymic acid), as well as phenolic acids (e.g., homogentisic acid) [18]. These compounds exert various pharmacological effects including anticancer, anti-inflammatory, antibacterial, and antioxidant activities. Current research shows that in CRC, the active constituents in ECD primarily affect pathways involved in tumorigenesis, immunization, and metabolism. Furthermore, experimental results indicate that ECD plays an important role in antiproliferation and promotion of apoptosis in CRC cells by downregulating the transcriptional levels of CDK1 and CDK6 and upregulating the transcriptional level of CDKN1A, thus inducing cell cycle arrest. As a compound medicine, ECD is effective in modulating multiple targets, not only directly regulating tumor cells but also affecting metabolism with other ingredients [19]. For example, Naringenin can reduce lipid peroxidation and protein carbonization, increase antioxidant defense, scavenge reactive oxygen species (ROS), and regulate fatty acid metabolism via signal pathways. These effects are conducive to fatty acid oxidation and the reduction of lipid accumulation [20], which may further affect tumor growth by altering lipid metabolism. The present study examined the lipid-lowering effects of ECD in mice that were fed an HF diet. ECD reduced the levels of GLU, TC, TG, and CRP indices and further alleviated inflammatory infiltration in HF-CRC mice. Further investigation into the mechanism of the lipid-lowering effects of ECD revealed that it downregulated ACSL4, ACSL1, CPT1A, and FASN levels in colorectal tissues and downregulated tumor formation in the colorectum. Taken collectively, the data suggest that ECD is an effective lipid-lowering compound that acted by regulating fatty acid metabolism.

The antitumor effects of ECD in combination with Huiyan Zhuyu decoction have been established in laryngeal carcinomas [21, 22]. ECD has also been used therapeutically for chronic bronchitis [23]. An obvious manifestation of colorectal inflammation-related diseases includes the shortening of colorectal length in mice [24]. It was found that HF-CRC aggravated this result, suggesting that the HF diet aggravated intestinal inflammation. Interestingly, colorectal length after ECD treatment was restored to improve inflammation. Furthermore, ECD was shown to improve insulin resistance and liver damage in rats, as manifested by serum aminotransferase levels and histopathological examination [25]. In daily clinical practice, lipid status is assessed based on serum concentrations of TC and TG [26]. In the present study, treatment with ECD significantly reduced GLU, TC, TG, and CRP levels and increased inflammatory infiltration in HF-CRC mice. The present study is partially in agreement with a previous study where ECD mitigated serum lipid and lipid transporter disorders in HFD-fed rats by elevating TG and TC [27]. Moreover, it was shown that the phlegm-resolving effects of ECD on HF mice were mainly evidenced by reduced body weight and levels of TG and TC [28].

To determine the detailed mechanism behind the effect of ECD on fatty acid metabolism, we measured fatty acids in the colorectal tissues of each group of mice. Studies have shown that *n* − 3 fatty acids suppress inflammation and *n* − 6 fatty acids promote inflammation [29, 30]. *N* − 3 and *n* − 6 polyunsaturated fatty acid supplementation resulted in the release of lipid mediators such as endocannabinoids, which are involved in food intake regulation, energy sensing, and food-related disorders [31]. Murad et al. found that docosahexaenoic acid application enhanced the antitumor effects of irradiation in CRC cells [32]. Moreover, meta-analysis showed that docosahexanoic acid and eicosapentaenoic acid were linked to an 11-12% reduced risk of CRC and linoleic acid to a 19% increased risk of CRC in patients of both sexes [33]. Our results were largely consistent with these published reports, suggesting that ECD accelerated the synthesis of *n* − 3 polyunsaturated fatty acids and inhibited the synthesis of *n* − 6 polyunsaturated fatty acids.

ACSLs, enzymes that catalyze the activation of long-chain fatty acids of 12–22 carbons, are major players in the synthesis of phospholipids and triacylglycerols [34]. More importantly, among the five family isoforms, ACSL1 and ACSL4 promote uncontrolled cell growth, facilitate tumor invasion, and lead to evasion of programmed cell death [35]. Furthermore, in rapidly proliferating tumor cells, the synthesis of fatty acids is accompanied by increased expression of major fatty acid synthesis enzymes including acetyl CoA carboxylase and FASN [36]. Overexpression of FASN is observed in multiple cancers and acts as a metabolic oncogene by modulating tumor growth and survival, making it a promising target for cancer therapy [37]. Schlaepfer et al. revealed that treatment with the irreversible CPT1 inhibitor etomoxir in nude mice contributed to decreased prostate tumor xenograft growth over 21 days, highlighting the therapeutic potential of impairing lipid catabolism to decrease prostate cancer tumor growth [38]. We also found that ECD effectively repressed the mRNA and protein expression of ACSL4, ACSL1, CPT1A, and FASN. Similar to our findings, Glavonoid-rich oil derived from an ethanol extract of licorice was found to decrease FASN expression in the inguinal white adipose tissues of diabetic mice [39]. Interestingly, ACSL1 overexpression using a recombinant adenoviral vector was associated with upregulated expression of FASN and elevated the proportion of eicosapentaenoic acid and polyunsaturated fatty acids in bovine adipocytes [40]. Nevertheless, there is little information available on the interactions among these four enzymes or the mechanisms of the main ingredients of ECD in regulating these enzymes in CRC. Future studies will investigate these questions.

The results of the present study demonstrate that ECD treatment inhibited inflammatory infiltration and fatty acid metabolism in CRC cells. ECD may exert its anticancer effects through regulation of fatty acid metabolism, but further research is needed to determine the underlying mechanisms. One potential weakness of this work is the lack of a negative control for ECD administration in mice. However, the findings derived from the present study combined with previous results shed light on the translational application of ECD in HF-related CRC.

## Figures and Tables

**Table 1:** List of fatty acid types, detection limits, and conversion coefficients of fatty acid methyl ester or fatty acid triglyceride to fatty acid (*Fi* or *Fj*).

Numbers	Fatty acids		Detection limit (mg/kg)	*Fi* conversion coefficient	*Fj* conversion coefficient
1	Caprylic acid	C8:0	0.5	0.9114	0.9192
2	Capric acid	C10:0	0.5	0.9247	0.9314
3	Undecanoic acid	C11:0	0.5	0.93	0.9363
4	Lauric acid	C12:0	0.5	0.9346	0.9405
5	Ficocerylic acid	C13:0	0.5	0.9386	0.9442
6	Myristic acid	C14:0	0.5	0.9421	0.9473
7	Myristoleic acid	C14:1n5	0.5	0.9417	0.947
8	Pentadecanoic acid	C15:0	0.5	0.9453	0.9502
9	Cis-10-pentadecanoic acid	C15:1n5	0.5	0.9449	0.9499
10	Hexadecanoic acid	C16:0	0.5	0.9481	0.9529
11	Palmitoleic acid	C16:1n7	0.5	0.9477	0.9525
12	Heptadecanoic acid	C17:0	0.5	0.9507	0.9552
13	Cis-10-heptadecanoic acid	C17:1n7	0.5	0.9503	0.9549
14	Octadecanoic acid	C18:0	0.5	0.953	0.9573
15	Elaidic acid	C18:1n9t	0.5	0.9527	0.957
16	Oleic acid	C18:1n9c	0.5	0.9527	0.9571
17	Linolelaidic acid	C18:2n6t	0.5	0.9524	0.9568
18	Linoleic acid	C18:2n6c	0.5	0.9524	0.9568
19	Arachidic acid;	C20:0	0.5	0.957	0.9609
20	Gamma-Linolenic acid	C18:3n6	0.5	0.952	0.9559
21	Eicosenoic acid	C20:1	0.5	0.9568	0.9608
22	*α*-linoleic acid	C18:3n3	0.5	0.952	0.956
23	Heneicosanoic acid	C21:0	0.5	0.9588	0.9628
24	Cis-11,14-eicosadienoic acid	C20:2	0.5	0.9565	0.9605
25	Behenic acid	C22:0	0.5	0.9604	0.9642
26	Cis-8,11,14-eicosatrienoic acid	C20:3n6	0.5	0.9562	0.9598
27	Erucic acid	C22:1n9	0.5	0.9602	0.9639
28	Cis-11,14,17-eicosatrienoic acid	C20:3n3	0.5	0.9562	0.9598
29	Arachidonic acid	C20:4n6	0.5	0.956	0.9597
30	Tricosanoic acid	C23:0	0.5	0.962	0.9658
31	Cis-13,16-docosadienoic acid	C22:2n6	0.5	0.96	0.9638
32	Lignoceric acid	C24:0	0.5	0.9963	1.0002
33	Cis-5,8,11,14,17-eicosapentaenoic acid	C20:5n3	1	0.9557	0.9592
34	Nervonic acid	C24:1n9	1	0.9632	0.9666
35	Cis-4,7,10,13,16,19-docosahexaenoic acid	C22:6n3	1	0.959	0.9624

*Note. Fi* is the conversion coefficient of fatty acid methyl ester into fatty acid; *Fj* is the conversion coefficient of fatty acid triglycerides into fatty acids.

**Table 2:** RT-qPCR primers.

Genes	Primers (5′⟶3′)
ACSL4-F	TCCTCCAAGTAGACCAACTCC
ACSL4-R	AATCCAGGTATGCGCTCACAC
ACSL1-F	TGCCAGAGCTGATTGACATTC
ACSL1-R	GGCATACCAGAAGGTGGTGAG
CPT1A-F	GGGTCGAAAGCCCATGTTGTA
CPT1A-R	CAGTGCTGTCATGCGTTGGA
Fas-F	AGCACTGCCTTCGGTTCAGTC
Fas-R	AAGAGCTGTGGAGGCCACTTG
GAPDH-F	GGTGAAGGTCGGTGTGAACG
GAPDH-R	CTCGCTCCTGGAAGATGGTG

*Note.* ACSL, acyl-CoA synthetase long-chain family member; CPT1A, carnitine palmitoyl transferase 1A; GAPDH, glyceraldehyde-3-phosphate dehydrogenase; F, forward; and R, reverse.aq.

## Data Availability

The data used to support the findings of this study are included in the article.

## Abbreviations

ECD: Erchen decoction
HF: High-fat
AOM/DSS: Azoxymethane/dextran sulphate sodium
GLU: Blood glucose; TC, total cholesterol; TG, total triglyceride
CRP: C-reactive protein
HE: Hematoxylin-eosin
RT-qPCR: Reverse transcription quantitative polymerase chain reaction
GAPDH: Glyceraldehyde-3-phosphate dehydrogenase
ACSL: Acyl-CoA synthetase long-chain family member
CPT1A: Carnitine palmitoyl transferase 1A
FASN: Fatty acid synthase.

## References

[1] Yarla N. S., Madka V., Pathuri G., Rao C. V. (2020). Molecular targets in precision chemoprevention of colorectal cancer: an update from pre-clinical to clinical trials. *International Journal of Molecular Sciences*.

[2] Jung G., Hernández-Illán E., Moreira L., Balaguer F., Goel A. (2020). Epigenetics of colorectal cancer: Biomarker and therapeutic potential. *Nature Reviews Gastroenterology & Hepatology*.

[3] Siegel R. L., Miller K. D., Goding Sauer A. (2020). Colorectal cancer statistics, 2020. *CA: A Cancer Journal for Clinicians*.

[4] McNabney S. M., Henagan T. M. (2017). Short chain fatty acids in the colon and peripheral tissues: a focus on butyrate, colon cancer, obesity and insulin resistance. *Nutrients*.

[5] Gallo G., Vescio G., De Paola G., Sammarco G. (2021). Therapeutic targets and tumor microenvironment in colorectal cancer. *Journal of Clinical Medicine*.

[6] Vaupel P., Multhoff G. (2021 Mar). Revisiting the Warburg effect: historical dogma versus current understanding. *The Journal of Physiology*.

[7] Ding C., Shan Z., Li M., Chen H., Li X., Jin Z. (2021). Characterization of the fatty acid metabolism in colorectal cancer to guide clinical therapy. *Molecular Therapy-Oncolytics*.

[8] Currie E., Schulze A., Zechner R., Walther T., Farese R. (2013). Cellular fatty acid metabolism and cancer. *Cell Metabolism*.

[9] Dierge E., Larondelle Y., Feron O. (2020). Cancer diets for cancer patients: Lessons from mouse studies and new insights from the study of fatty acid metabolism in tumors. *Biochimie*.

[10] Yan Z., Lai Z., Lin J. (2017). Anticancer properties of traditional Chinese medicine. *Combinatorial Chemistry & High Throughput Screening*.

[11] Gao B. Z., Chen J. C., Liao L. H., Xu J. Q., Lin X. F., Ding S. S. (2015). Erchen decoction prevents high-fat diet induced metabolic disorders in C57BL/6 mice. *Evidence-based Complementary and Alternative Medicine*.

[12] Jiang Y., Fan L. (2021). The effect of Poria cocos ethanol extract on the intestinal barrier function and intestinal microbiota in mice with breast cancer. *Journal of Ethnopharmacology*.

[13] Lin T. Y., Lu M. K., Chang C. C. (2020). Structural identification of a fucose-containing 1, 3-*β*-mannoglucan from Poria cocos and its anti-lung cancer CL1-5 cells migration via inhibition of TGF*β*R-mediated signaling. *International Journal of Biological Macromolecules*.

[14] Jiang Y., Fan L. (2020). Evaluation of anticancer activities of Poria cocos ethanol extract in breast cancer: in vivo and in vitro, identification and mechanism. *Journal of Ethnopharmacology*.

[15] Zhang Z., Yang L., Hou J., Tian S., Liu Y. (2021). Molecular mechanisms underlying the anticancer activities of licorice flavonoids. *Journal of Ethnopharmacology*.

[16] Snider A. J., Bialkowska A. B., Ghaleb A. M., Yang V. W., Obeid L. M., Hannun Y. A. (2016). Murine model for Colitis-associated cancer of the colon. *Methods in Molecular Biology*.

[17] Saetang J., Sangkhathat S. (2017). Diets link metabolic syndrome and colorectal cancer development. *Oncology Reports*.

[18] Yang H. J., Yim N. H., Lee K. J. (2016). Simultaneous determination of nine bioactive compounds in Yijin-tang via high-performance liquid chromatography and liquid chromatography-electrospray ionization-mass spectrometry. *Integrative Medicine Research*.

[19] Shao Y., Chen J., Hu Y., Wu Y., Zeng H., Sun J., Zhou X., Lai Q., Fan X. (2022 Oct 13). Investigating the effects and mechanisms of Erchen Decoction in the treatment of colorectal cancer by network pharmacology and experimental validation. *Frontiers in Pharmacology*.

[20] Zobeiri M., Belwal T., Parvizi F. (2018). Naringenin and its Nano-formulations for fatty liver: Cellular Modes of action and clinical Perspective. *Current Pharmaceutical Biotechnology*.

[21] Zhou S., Luo Q., Tan X. (2020). Erchen decoction plus huiyanzhuyu decoction inhibits the cell cycle, migration and invasion and induces the apoptosis of laryngeal squamous cell carcinoma cells. *Journal of Ethnopharmacology*.

[22] Tan X., Luo Q., Zhou S. (2020). Erchen plus huiyanzhuyu decoction inhibits the growth of laryngeal carcinoma in a mouse model of phlegm-Coagulation-blood-Stasis syndrome via the STAT3/Cyclin D1 pathway. *Evidence-based Complementary and Alternative Medicine*.

[23] Chen L. P., Cai Y. M., Li J. S. (2017). Medication rules of famous veteran traditional Chinese medicine doctor in treatment of chronic bronchitis based on implicit structure model. *China Journal of Chinese Materia Medica*.

[24] Zhang W., Zou G., Li B. (2020 Aug 28). Fecal microbiota Transplantation(FMT) Alleviates experimental colitis in mice by gut microbiota regulation. *Journal of Microbiology and Biotechnology*.

[25] Zhang H., Ta N., Chen P., Wang H. (2017). Erchen decoction and linguizhugan decoction ameliorate hepatic insulin resistance by inhibiting IRS-1Ser307 phosphorylation in vivo and in vitro. *Evidence-based Complementary and Alternative Medicine*.

[26] Pakiet A., Kobiela J., Stepnowski P., Sledzinski T., Mika A. (2019). Changes in lipids composition and metabolism in colorectal cancer: a review. *Lipids in Health and Disease*.

[27] Ding S., Kang J., Tong L., Lin Y., Liao L., Gao B. (2018). Erchen decoction ameliorates lipid metabolism by the regulation of the protein CAV-1 and the receptors VLDLR, LDLR, ABCA1, and SRB1 in a high-fat diet rat model. *Evidence-based Complementary and Alternative Medicine*.

[28] Zhang M., Shao Y., Gao B. (2020). Erchen decoction mitigates lipid metabolism disorder by the regulation of PPAR*γ* and LPL gene in a high-fat diet C57BL/6 mice model. *Evidence-based Complementary and Alternative Medicine*.

[29] Michalak A., Mosińska P., Fichna J. (2016). Polyunsaturated fatty acids and their Derivatives: therapeutic value for inflammatory, Functional Gastrointestinal disorders, and colorectal cancer. *Frontiers in Pharmacology*.

[30] Del Cornò M., Baldassarre A., Calura E. (2019). Transcriptome Profiles of human Visceral adipocytes in obesity and colorectal cancer Unravel the effects of body mass Index and polyunsaturated fatty acids on genes and Biological processes related to tumorigenesis. *Frontiers in Immunology*.

[31] D’Angelo S., Motti M. L., Meccariello R. (2020). *ω*-3 and *ω*-6 polyunsaturated fatty acids, obesity and cancer. *Nutrients*.

[32] Murad L. B., da Silva Nogueira P., de Araújo W. M. (2019). Docosahexaenoic acid promotes cell cycle arrest and decreases proliferation through WNT/*β*‐catenin modulation in colorectal cancer cells exposed to *γ*‐radiation. *BioFactors*.

[33] Nguyen S., Li H., Yu D. (2021). Dietary fatty acids and colorectal cancer risk in men: a report from the Shanghai Men’s Health Study and a meta‐analysis. *International Journal of Cancer*.

[34] Rossi Sebastiano M., Konstantinidou G. (2019). Targeting long chain acyl-coa synthetases for cancer therapy. *International Journal of Molecular Sciences*.

[35] Tang Y., Zhou J., Hooi S. C., Jiang Y. M., Lu G. D. (2018). Fatty acid activation in carcinogenesis and cancer development: Essential roles of long-chain acyl-CoA synthetases. *Oncology Letters*.

[36] Tang Y., Tang R., Tang M. (2020). LncRNA DNAJC3-AS1 regulates fatty acid synthase via the EGFR pathway to promote the progression of colorectal cancer. *Frontiers in Oncology*.

[37] Flavin R., Peluso S., Nguyen P. L., Loda M. (2010). Fatty acid synthase as a potential therapeutic target in cancer. *Future Oncology*.

[38] Schlaepfer I. R., Rider L., Rodrigues L. U. (2014). Lipid catabolism via CPT1 as a therapeutic target for prostate cancer. *Molecular Cancer Therapeutics*.

[39] Igarashi Y., Iida S., Dai J. (2021). Glavonoid-rich oil supplementation reduces stearoyl-coenzyme A desaturase 1 expression and improves systemic metabolism in diabetic, obese KK-A mice. *Biomedicine & Pharmacotherapy*.

[40] Zhao Z., Raza S. H. A., Tian H. (2020). Effects of overexpression of ACSL1 gene on the synthesis of unsaturated fatty acids in adipocytes of bovine. *Archives of Biochemistry and Biophysics*.

